# Massive stocking of chum salmon (*Oncorhynchus keta*) fry fattens non-native brown trout (*Salmo trutta*) in Hokkaido, Japan

**DOI:** 10.1371/journal.pone.0307552

**Published:** 2024-07-19

**Authors:** Kentaro Honda, Koh Hasegawa, Masatoshi Ban, Yutaka Yano, Yuhei Ogura

**Affiliations:** Salmon Research Department, Fisheries Resources Institute, Japan Fisheries Research and Education Agency, Sapporo, Hokkaido, Japan; CIFRI: Central Inland Fisheries Research Institute, INDIA

## Abstract

In Japan, stocked chum salmon (*Oncorhynchus keta*) fry may have become the perfect prey for non-native brown trout (*Salmo trutta*), which are popular targets of anglers. If this is the case, fry stocking which is intended to boost commercial fishing may be helping to sustain the populations of an invasive predator. We used dietary and biochemical analyses to examine whether brown trout quickly restore their nutritional status following wintertime declines by preying upon chum salmon fry that are stocked in spring. We targeted six rivers in Hokkaido, Japan, three with fry stocking and three without. Changes in brown trout condition factor, triglyceride contents in muscle and serum, serum insulin-like growth factor-1 (IGF-1; an indicator of short-term growth), and docosahexaenoic acid (DHA; an essential fatty acid abundant in fish) content in muscle were examined between before stocking and during the stocking period in the six rivers. Dietary analysis showed that brown trout preyed on fry during the stocking period in all stocked rivers. Their nutritional status tended to be higher during the stocking period than before stocking in stocked rivers, but not in unstocked rivers. These results suggest that the massive stocking of chum salmon fry provides brown trout with the perfect prey to quickly restore their nutritional status and fuel increased growth; this may therefore be a controversial issue among stakeholders.

## Introduction

Freshwater systems are closely connected to human life. They are important places of food production and host both wild-catch and aquaculture fisheries [1‒3]. Additionally, they are popular destinations for recreation [4], with recreational fishing being one of the most popular activities worldwide, with economic impacts [5]. Consequently, both native and non-native fish species have been stocked into freshwater systems to enhance fisheries resources for both commercial and recreational purposes [1, 6, 7]. In South Africa, for example, common carp (*Cyprinus carpio*), salmonids, and centrarchids (all non-native to the region) have been introduced to inland waters for angling and food production since the late 1800s [8]. These stocked fishes have caused declines of populations of other sympatric species through interspecific interactions such as competition and predation [8]. Thus, conflicts have arisen between people with differing opinions on fish stocking.

Brown trout (*Salmo trutta*) are a popular recreational fishing target worldwide, both in and out of their native range [9]. This species is also listed on the IUCN’s “100 of the World’s Worst Invasive Alien Species” [10], and there are considerable concerns about their negative impacts on invaded regions [9]. In Japan, brown trout are believed to have been first introduced in 1892; since then, they have been widely transplanted across Hokkaido and are now found in many rivers in northern Japan [11, 12]. In the process, brown trout populations in Hokkaido have replaced native white-spotted charr (*Salvelinus leucomaenis*) [13, 14]. In the Falkland Islands, brown trout have been reported to expand their distribution through the movement of anadromous individuals in the ocean [15]. In Japan, the presence of anadromous individuals has been confirmed in several rivers [16, 17], and an individual with anadromous life history has recently been identified in a small river in southern Hokkaido, where the presence of brown trout had not previously been confirmed [18]. Thus, the presence and range expansion of non-native brown trout are feared to have ecosystem-level impacts [9] and to seriously affect native ecosystems in Japan [11, 12, 19].

In the North Pacific, Pacific salmon (*Oncorhynchus* spp.) are an important fishery resource and have economic value. They are stocked widely through stock enhancement programs, contributing greatly to commercial fisheries resources [20]. However, salmon stocking does not simply increase the abundance of the target species: It also carries the risk of negative impacts on the wider ecosystem at the stocked site, such as by reducing wild populations of the same species or populations of interacting species [21‒24]. Hasegawa et al. [25] showed that stocked chum salmon (*Oncorhynchus keta*) fry decreased the foraging efficiency and growth of wild native masu salmon (*O*. *masou*) fry through interspecific competition and reduced the abundance of aquatic invertebrates through predation.

Chum salmon are native to Japan; as the species most commonly stocked over a wide region, they are a significant contributor to commercial fishery resources in Japan [20, 26]. Most chum salmon fry stocked in spring migrate to the sea within a short period (mostly within a month in Japan, where the distances from stocking points to the sea are less than 100 km) [27]. Chum salmon suffer massive mortality during their early life history [26], likely including by predation in rivers [28‒30], as has been observed in salmon juveniles and fry in other regions [e.g., 31‒33]. Although reducing this predation pressure is desirable to protect stocked salmon fry, it is not realistic to artificially reduce mortality from predators that are widely distributed in both rivers and oceans, unless the removal of predators is judged to be justified.

Because brown trout prey upon chum salmon fry, local commercial fishermen consider brown trout to be harmful to their operations [19]. On the other hand, recreational anglers that target brown trout regard this predation on chum salmon fry to be an inconvenient fact that they fear could provide an excuse to justify a removal program [34]. Nevertheless, even if brown trout were eliminated, native fishes such as white-spotted charr would still prey on fry [30]. Therefore, we believe that the initial focus should not be on reducing the total number of fry preyed upon, but on building consensus between commercial fishermen and recreational anglers to properly manage rivers where hatchery-raised chum salmon and brown trout coexist [19]. To do so, understanding the predator–prey interactions between stocked chum salmon fry and introduced brown trout is essential, but little research on this has been conducted to date.

Brown trout have been observed to individually prey on >100 chum salmon fry in the Chitose River, Hokkaido, within 1 day after fry stocking [29]. Additionally, brown trout in the river do not show noticeable seasonal migration associated with chum fry stocking [29]. In many stocked rivers in Hokkaido, stocking is repeated over multiple days in spring, and the total stocking number exceeds 1 million (Fisheries Resources Institute, unpublished data). These data suggest that the consumption of stocked fry might contribute to brown trout survival and growth. In particular, because the brown trout spawning season in Hokkaido is from autumn to winter [12, 35], brown trout in spring are at their most exhausted after reproduction and overwintering. Moreover, the post-spawning period in fish is generally considered to be a period of accelerated growth [36]. Therefore, the massive stocking of fry in this season could provide brown trout with the perfect food supply in terms of both quantity and quality to restore their depleted nutritional status. If this is the case, then fry stocking which is intended to boost commercial fishing may contribute to sustaining the populations of an invasive predator. Recently, it was reported that concentrations of essential fatty acids in predatory white-spotted charr were increased by predation on stocked masu salmon fry in which the fatty acid content had been enhanced by the feeding of artificial pellets [37]. The same mechanism may be occurring in invasive brown trout. The objective of the present study was to use dietary and biochemical analysis of body tissues to test the hypothesis that brown trout quickly restore their nutritional status following wintertime declines by preying on stocked chum salmon fry.

## Materials and methods

### Study site

**Fig 1:**
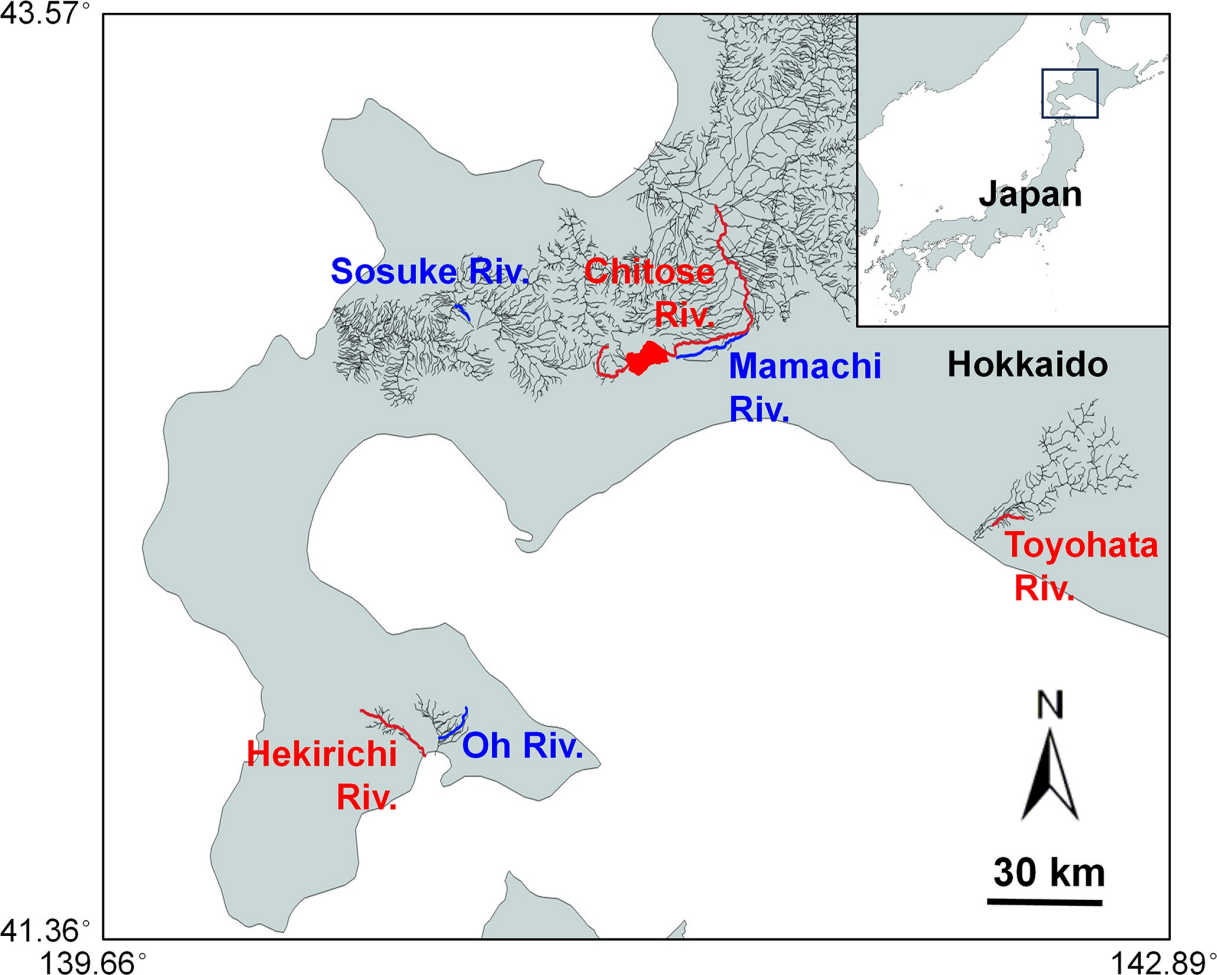
Map of the study site. Red (blue) lines show rivers where chum salmon fry are stocked (unstocked). See Table 1 for detailed sampling locations in each river. River data: Reprinted under a CC BY license, with permission from Information Utilization Division, Real Estate and Construction Economy Bureau, MLIT of Japan, original copyright 2009. Maps were made with Natural Earth. Free vector and raster map data @ naturalearthdata.com.

A total of six rivers in central and southern Hokkaido were selected as study sites (Fig 1). Brown trout are common in these rivers [17, 29, 35, 38]. Three of the selected rivers are regularly stocked with chum salmon fry: (1) the Hekirichi River, (2) the main stem of the Chitose River in the Ishikari River system, and (3) the Toyohata River in the Shizunai River system. In these rivers, large releases of hatchery-raised chum salmon fry are conducted multiple times over a 2-month period annually. In 2022, a total of 5.91 million fry (mean fork length [FL]: 5.2 cm) were stocked in the Hekirichi River during 10 releases between 11 March and 18 April, 30.29 million fry (4.5 cm FL) in the Chitose River during 8 releases between 3 March and 23 April, and 12.43 million fry (6.7 cm FL) in the Toyohata River during 14 releases between 11 April and 26 May (S1 Table in S1 File; Fisheries Resources Institute, unpublished data). The other three selected rivers were unstocked: (4) the Oh River in the Kunebetsu River system, (5) the Sosuke River in the Shiribetsu River system, and (6) the Mamachi River in the Chitose River system, all of which are connected to the sea and are capable of supporting wild chum salmon runs. However, no chum salmon adults or fry were observed during the study period in any of the three unstocked rivers.

The introduction history of brown trout to these rivers is not clear. The year of the first recorded brown trout appearance is known in some rivers (e.g., 1984 in the Chitose River [39]), but the number of fish initially stocked and the persons who conducted brown trout stocking are unknown. Takami and Aoyama [40] have speculated that private anglers stocked brown trout into rivers in Hokkaido, but stocking of brown trout is prohibited by the Hokkaido government at present.

### Fish sampling

In the Chitose and Mamachi rivers, brown trout were sampled once a month from February to June 2022, including in March and April, when chum salmon fry were stocked in the Chitose River (Table 1; S1 Table in S1 File). In other rivers, sampling was conducted once before (January‒February) and once during the peak (April‒May) of the fry stocking period in 2022. Sampling periods for each unstocked river were matched to those for the nearest stocked river. A total of 9‒15 fish were collected in each river during each sampling period by using an electrofisher (model 12B; Smith Root Co., Vancouver, WA, USA), except for 2 fish collected by angling in May in the Hekirichi River (Table 1). Although the last fry release in the Hekirichi River occurred on 18 April, sampling for the peak stocking period was delayed to 7 May because of increased snowmelt in April, which made sampling impossible. The collected fish were immediately euthanized by cranial pithing and were frozen at –30°C or below until analysis.

**Table 1:** Summary of brown trout sampling.

River	Stocking status of chum salmon fry	Before or at peak of the stocking period^1^	Capture date (in 2022)	Capture area range (north latitude, east longitude)	No. captured fish	Fork length (cm)
Hekirichi	Stocked	Before	17 Jan	41.8202°, 140.6493°‒ 41.8377°, 140.6370°	15	21.6‒39.5
		Peak^2^	7 May		10	14.0‒39.9
Chitose	Stocked	Before	17 Feb	42.8277°, 141.6589°‒ 42.8092°, 141.5758°	13 (12)	11.8‒51.8
		-	18 Mar		13 (10)	11.4‒62.7
		Peak	12 Apr		12 (10)	14.0‒33.5
		-	13 May		12 (11)	14.8‒61.1
		-	15 Jun		12 (12)	18.2‒30.5
Toyohata	Stocked	Before	14 Feb	42.3662°, 142.4266°‒ 42.3839°, 142.4546°	9	10.3‒63.5
		Peak	19 May		10	13.2‒57.2
Oh	Unstocked	Before	16 Jan	41.8618°, 140.7024°‒ 41.8619°, 140.7047°	11	22.6‒44.5
		Peak	18 Apr		13	15.7‒41.5
Sosuke	Unstocked	Before	15 Feb	42.8812°, 140.7508°‒ 42.8822°, 140.7544°	13	12.3‒51.8
		Peak	5 Apr		11	12.7‒35.7
Mamachi	Unstocked	Before	17 Feb	42.7926°, 141.5901°‒ 42.7870°, 141.5869°	15 (10)	14.3‒36.3
		-	18 Mar		12 (9)	14.5‒29.1
		Peak	12 Apr		12 (10)	18.5‒25.8
		-	13 May		11 (11)	17.4‒27.4
		-	15 Jun		11 (11)	17.9‒27.9

Numbers in parentheses indicate the numbers of individuals sampled for blood. ^1^ Sampling periods for unstocked rivers matched those of the nearest stocked river. ^2^ Sampling in the peak period (April) in the Hekirichi River was delayed because of snowmelt.

In the Chitose and Mamachi rivers, among the fish collected, blood samples from 9‒12 fish per month were collected from the caudal peduncle on site by using a syringe (2.5 mL) for analysis of blood composition (Table 1). Blood was injected into 1.5-mL tubes without an anticoagulant, kept cold below 10°C, and brought back to the laboratory. Blood was centrifuged (1000 ×*g*, 15 min) on the same day, and the supernatant serum was collected and stored at –80°C until analysis.

A total of 215 brown trout were analyzed, with an FL range (mean ± SD) of 10.3‒63.5 cm (24.1 ± 10.2 cm), which was measured after thawing (Table 1; S2 Table in S1 File). In this study, we targeted brown trout of size 10 cm FL or larger because brown trout of 8.7 cm FL have been found to prey on masu salmon fry (which are very similar in body shape to chum salmon fry) in the Chitose River [41].

### Dietary analysis

Brown trout were thawed and sexed and measured for FL and body weight (BW, g). Stomach contents were then removed from the excised stomachs and identified to the species level whenever possible. Classified prey items were counted and their wet weight was measured to the nearest 0.001 g. Because the main objective of the present study was to understand the effects of predation on chum salmon fry, fish were classified as either chum salmon fry or as “other fishes.” Other prey items varied and therefore were categorized mainly at the order level as fish eggs, Ezo brown frog (*Rana pirica*), Amphipoda, Decapoda, Ephemeroptera, Plecoptera, Megaloptera, Trichoptera, Diptera, Gastropoda, other aquatic invertebrates, terrestrial invertebrates, and others. Given the variation in body size of specimens among groups in each river and month, the mean wet weight (MWW) per 100-g converted fish for each prey taxon (excluding digestate) was determined by reference to Tadokoro et al. [42], by using the following formula:

$$MWW=\frac{1}{n}\sum_{i=1}^{n}\frac{W_{i}}{{BW}_{Ti}}\times 100,$$ (1)

where *W*_*i*_ is the wet weight of a prey taxon preyed upon by individual *i*, *n* is the number of predator specimens in each river and month, and BW_T*i*_ is the true body weight of individual *i*, excluding the total wet weight of stomach contents. MWW was calculated for all prey taxa, and the dominance of prey taxa was compared among groups.

### Biochemical analysis

Fish condition factor (CF) was determined from the following equation:

$$CF=\frac{{BW}_{T}}{{FL}^{3}}\times 1000,$$ (2)

where BW_T_ is the true body weight. In fish, neutral lipids represented by triglycerides (TG) are taken up into the blood as an important energy source and subsequently stored in body tissues such as muscle and liver [43]. Therefore, TG concentrations in blood and muscle are often used as indicators of nutritional status in salmonids [44‒47]. Chum salmon fry just before stocking are reported to have higher TG values than wild fry [48]. Additionally, docosahexaenoic acid (DHA) is an *n*-3 polyunsaturated fatty acid that is abundant in fish, including salmonids, making it a reasonable lipid constituent for assessing the effects of predation on salmon fry [37, 49]. Freshwater salmonids retain DHA directly from their food and also synthesize it from precursors such as eicosapentaenoic acid (EPA), whereas invertebrates such as insects are rich in EPA but poor in DHA as a result of the types of food they consume and their limited ability to synthesize DHA [50]. Serum insulin-like growth factor-1 (IGF-1) is a known indicator of short-term growth and has recently been used for this purpose in salmonids [51, 52]. In the present study, muscle TG content (M-TG, mg/g), serum TG concentration (S-TG, mg/dL), muscle DHA content (mg/g), and IGF-1 concentration (ng/mL) were used as indicators of nutritional status in addition to CF.

M-TG was measured by using LabAssay Triglyceride (GPO-DAOS method; FUJIFILM Wako Pure Chemical Corporation, Osaka, Japan) following Torao [47, 53]. About 2 g of half-thawed dorsal muscle was excised, and lipids were extracted with 10 times the volume of ethanol-ether (3:1) solution. This was followed by centrifugation (1300 ×*g*, 20 min) to remove foreign substances. The supernatant liquid was mixed with a chromogenic solution, soaked in 37°C water for 5 min, and measured by colorimetric analysis at a wavelength of 630 nm.

S-TG and IGF-1 were analyzed for all specimens sampled for blood in the Chitose and Mamachi rivers. For S-TG, 5 μL was taken from serum dissolved on ice and measured in the same manner as the supernatant of M-TG. IGF-1 was measured by using an IGF-1 ELISA Kit (Cusabio Technology LLC., Houston, TX, USA). Microplates coated with IGF-1 antibody were loaded with 10 μL of dissolved serum and horseradish peroxidase and allowed to react at 37°C for 1 h. A chromogenic solution was added, and the reaction was carried out at 37°C for 15 min before colorimetric analysis at a wavelength of 450 nm.

The analysis of fatty acids was conducted as described by Hasegawa et al. [37]. Seven fish each were randomly selected for analysis before stocking and during the peak of the stocking period in each river. A piece of tissue and skin was sampled from defrosted brown trout from the area between the gill cover and dorsal fin for lipid extraction. Total lipids were extracted by using the method described by Folch et al. [54]. The total lipids were quantitatively transferred to a reaction tube and added to 406 μg of docosanoic acid (22:0) as an internal standard. Fatty acid methyl esters were prepared by acid-catalyzed methylation by using the procedure described by Ichihara and Fukubayashi [55] and analyzed by using gas chromatography (8860GC; Agilent, Santa Clara, CA, USA) with an Omegawax 320 capillary column (length 30 m, internal diameter 0.32 mm, phase thickness 0.25 um) (Sigma-Aldrich Co., St. Louis, MO, USA) and a flame ionization detector. Helium was used as the carrier gas. Chromatograms were integrated by using Agilent OpenLAB data analysis software. Fatty acid methyl esters were identified by comparison of the retention times with those of known standards (Sigma-Aldrich Co.). Quantification of DHA was conducted by using the ratio of the DHA peak areas to the internal standard peak area (22:0).

### Statistical analysis

#### Comparison between the Chitose and Mamachi rivers

R ver. 4.2.2 [56] was used for all statistical analyses. Indices of nutritional status (CF, M-TG, S-TG, and IGF-1) obtained in each month were compared between the Chitose and Mamachi rivers. General linear models were constructed with CF or log_10_-transformed M-TG, S-TG, or IGF-1 as objective variables and log_10_FL (excluded from the CF model), river (Chitose or Mamachi), month (February to June, ordered as categorical variables), and the interaction of river and month as explanatory variables, as follows:

CF=river+month+river×month (3)

$$log_{10}M‐TG,\;log_{10}S‐TG,\;or\;log_{10}IGF‐1=log_{10}FL+river+month+river\times month.$$ (4)

Model outputs were analyzed with Type III ANOVA by using the *Anova* function in the package “car” [57]. To determine the effect of FL on CF, we examined the relationship between FL and BW_T_ in each month by using a general linear model:

$$log_{10}BW_{T}=log_{10}FL+river+log_{10}FL\times river.$$ (5)

If the interaction term log_10_FL × river was significant (*ɑ* = 0.05), this was interpreted as indicating a difference in the slope of the regression line between rivers.

#### Comparison between pre- and peak stocking periods

To determine if the trends observed in the Chitose and Mamachi rivers were consistent with those in other rivers, we added DHA as a factor (while excluding S-TG and IGF-1) and examined whether CF, M-TG, and DHA varied between the pre- and peak stocking periods for chum salmon fry in the six rivers. Linear mixed models with the difference between rivers as a random effect were constructed by using the package “lmerTest” [58], with CF, log_10_-transformed M-TG, or DHA as objective variables and log_10_FL (except in the CF model), timing (pre- or peak stocking period), river type (RT; stocked or unstocked), and the interaction of timing and RT as explanatory variables, as follows:

CF=timing+RT+timing×RT (6)

$$log_{10}M‐TG\;or\;log_{10}DHA=FL+timing+RT+timing\times RT.$$ (7)

Model outputs were analyzed with Type III ANOVA. To determine the effect of FL on CF, we examined the relationship between FL and BW_T_ by using the following general linear model:

$$log_{10}BW_{T}=log_{10}FL+RT+log_{10}FL\times RT.$$ (8)

Separate models were constructed for pre- and peak stocking periods. If the interaction term log_10_FL × RT was significant (*ɑ* = 0.05), this was interpreted as indicating a difference in the slope of the regression line between river types. Finally, because DHA is a component of M-TG, correlations between the three indices (including CF) were tested by using Pearson’s correlation test (*ɑ* = 0.05) between stocked and unstocked rivers before and at the peak of the stocking periods.

### Ethics statement

The authors obtained legal permission from the governor of Hokkaido Prefecture for fish sampling and handling (permit numbers 158, 159, 161, and 184 for sampling organisms in inland waters). Permission for animal experiments was not required from the Japan Fisheries Research and Education Agency. All methods were carried out in accordance with the ARRIVE guidelines ver. 2.0 [59]. The collected fish were immediately euthanized by cranial pithing.

## Results

### Dietary analysis

**Fig 2:**
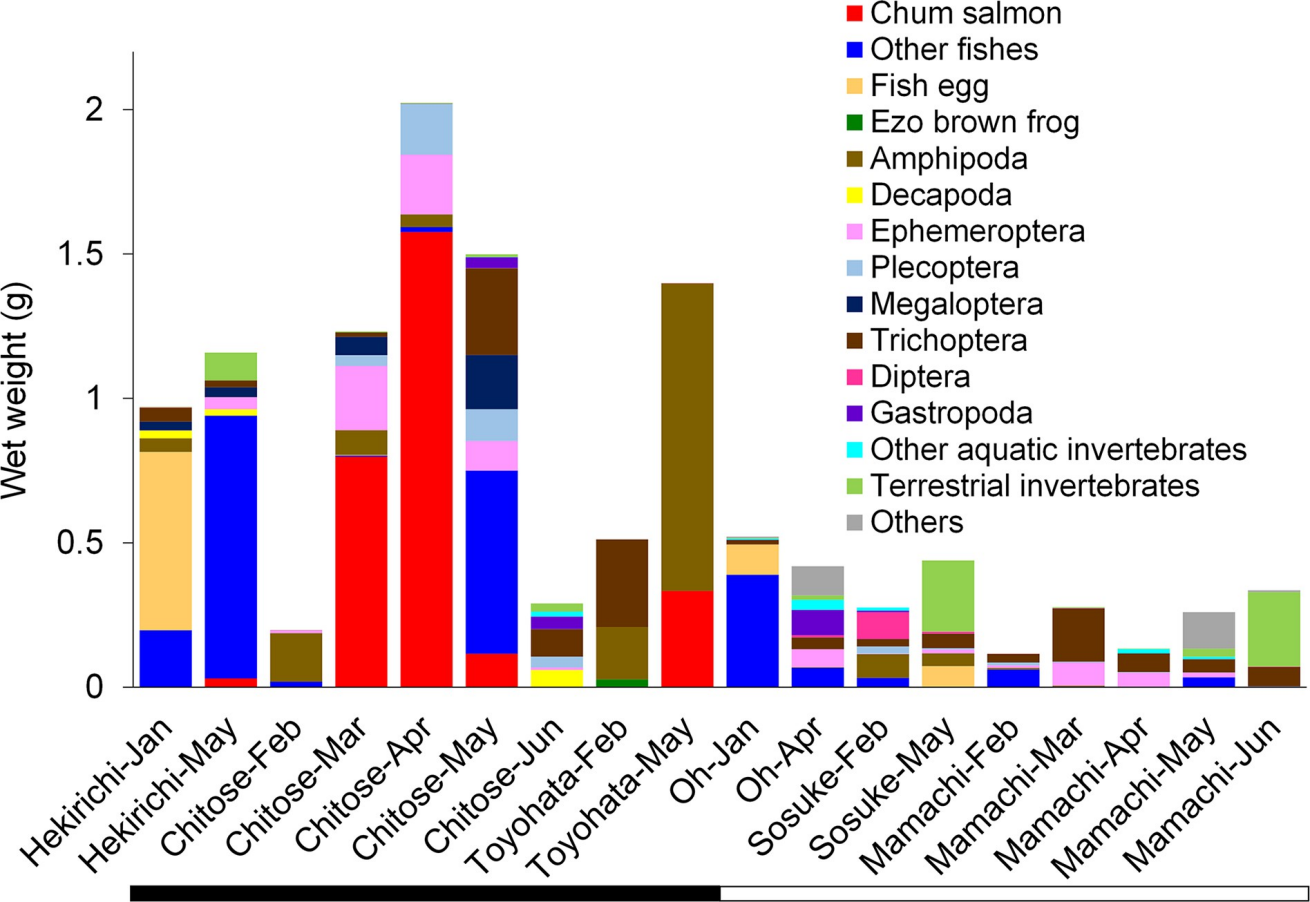
Mean wet weight per 100-g converted brown trout for each stomach content category in each river and month. Black and white bars show stocked and unstocked rivers, respectively.

Chum salmon fry were detected in the stomachs of brown trout collected in all stocked rivers during the peak of the stocking period (Fig 2; S2 Table in S1 File). Fry were additionally found in March (during the stocking period) and May (after the stocking period) in the Chitose River, with particularly high MWW in March and April. Four, six, and two brown trout preyed on 1‒97 chum salmon fry in the Chitose River in March, April, and May, respectively. One and three brown trout in the Hekirichi and Toyohata rivers preyed on 1 and 1‒3 fry, respectively. Mean FL ± SD (range) of brown trout that fed on fry was 31.3 ± 14.8 cm (15.0‒62.7 cm). Predation on chum salmon fry was not observed in other rivers or months. During the peak of the stocking period in the Hekirichi and Toyohata rivers, other fishes (ice goby *Leucopsarion petersii*, and *Gymnogobius* spp.) and Amphipoda, respectively, accounted for a relatively high proportion of the stomach contents not taken up by chum salmon fry (Fig 2). Total MWW was lower in unstocked rivers, where aquatic and terrestrial invertebrates were dominant.

### Biochemical analysis

#### Comparison between the Chitose and Mamachi rivers

**Fig 3:**
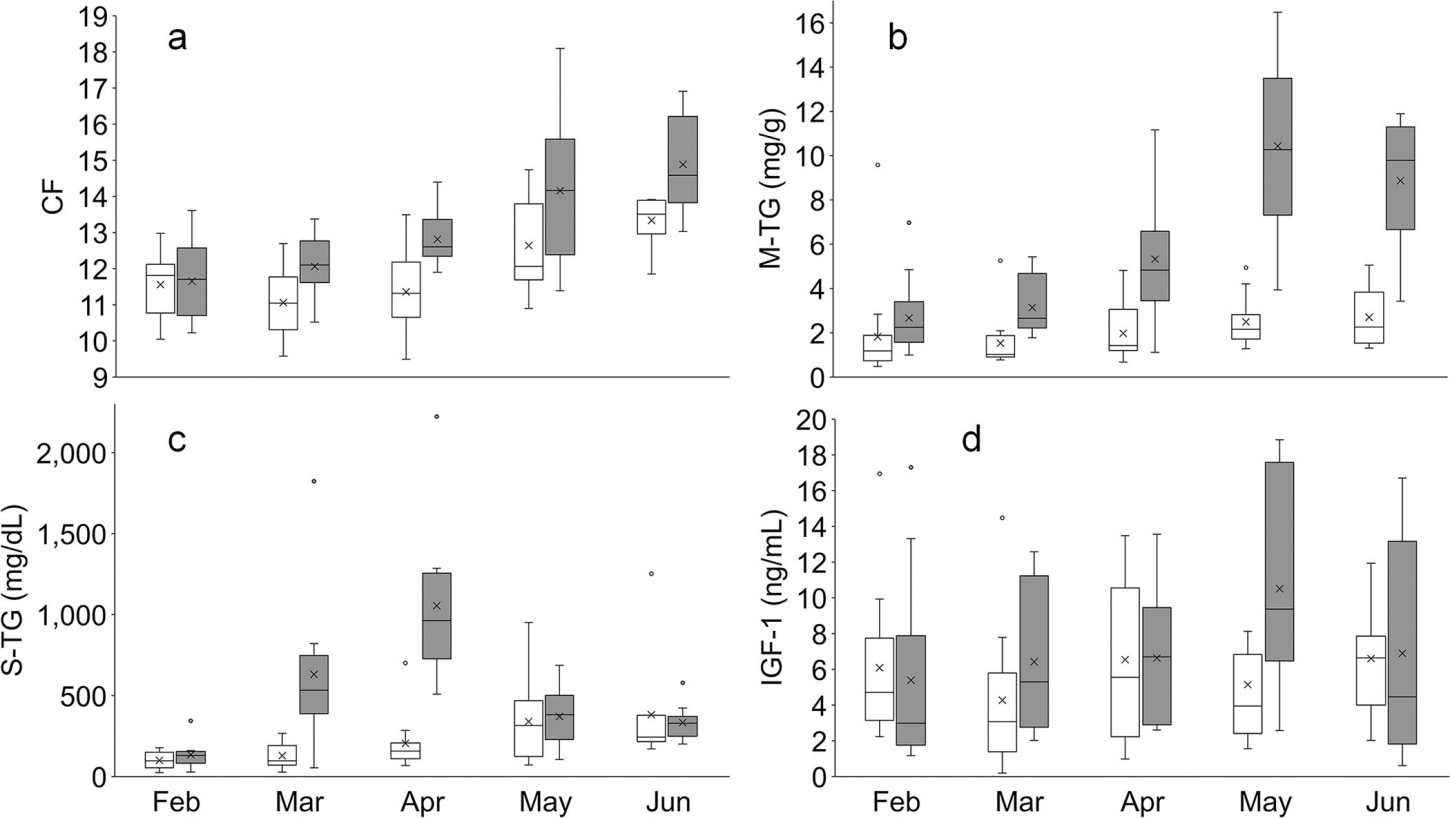
Box plots of (a) condition factor (CF), (b) triglyceride in muscle (M-TG) and (c) serum (S-TG), and (d) serum insulin-like growth factor-1 (IGF-1) for brown trout sampled from the Mamachi (white) and Chitose (gray) rivers from February to June 2022. Chum salmon fry were stocked in March and April in the Chitose River. Crosses and lines in the middle of boxes show means and medians. Box upper and lower boundaries show 75th and 25th percentiles, respectively. Bars extend to 1.5 times the box height above and below the boxes. Small circles show outlier data points.

In the general linear model for CF, the variables river and month were significant, showing that CF was higher in the Chitose River than in the Mamachi River and that CF increased as the months progressed (Table 2; Fig 3A). Although the interaction term was insignificant, CF had similar values between the two rivers only in February (before fry stocking). The slope of log_10_FL relative to log_10_BW_T_ did not differ between rivers in any month (i.e., log_10_FL × river was not significant, *F* < 1.25, *p* > 0.277; S1 Fig in S2 File). River × month was significant in the M-TG model, indicating that M-TG tended to increase more over the months in the Chitose River than in the Mamachi River, reaching a maximum in May (Table 2; Fig 3B). River × month was also significant in the S-TG model, reflecting the higher S-TG in the Chitose River than in the Mamachi River during March and April (Table 2; Fig 3C). S-TG in the Chitose River then dropped sharply in May and became comparable to that in the Mamachi River. River × month had a marginal effect in the IGF-1 model, which showed a large difference between rivers in May (Table 2; Fig 3D). FL was not significant in the M-TG and IGF-1 models but had a negative effect on S-TG (estimated coefficient = –0.491; Table 2). Extremely high S-TG exceeding 1000 mg/dL was observed in a total of seven fish with relatively small FL (17.1‒22.6 cm).

**Table 2:** Results of general linear models testing the effects of log_10_-transformed fork length (log_10_FL), river (Chitose or Mamachi), month (February‒June), and an interaction term (river × month) on brown trout condition factor (CF), triglyceride in muscle (log_10_M-TG) and serum (log_10_S-TG), and insulin-like growth factor-1 (log_10_IGF-1).

	Sum of squares	d.f.	*F*	*p*
**CF**				
River	38.4	1	31.17	<0.001
Month	93.9	4	19.06	<0.001
River × month	10.1	4	2.04	0.093
**Log**_**10**_ **M-TG**				
Log_10_FL	0.10	1	1.88	0.174
River	6.03	1	115.27	<0.001
Month	3.85	4	18.38	<0.001
River × month	0.59	4	2.84	0.028
**Log**_**10**_ **S-TG**				
Log_10_FL	0.37	1	5.15	0.025
River	3.04	1	41.92	<0.001
Month	4.83	4	16.63	<0.001
River × month	2.50	4	8.61	<0.001
**Log**_**10**_ **IGF-1**				
Log_10_FL	0.07	1	0.60	0.440
River	0.20	1	1.65	0.202
Month	0.92	4	1.86	0.124
River × month	1.07	4	2.17	0.078

Chum salmon fry were stocked in the Chitose River in March and April.

#### Comparison between pre- and peak stocking periods

**Fig 4:**
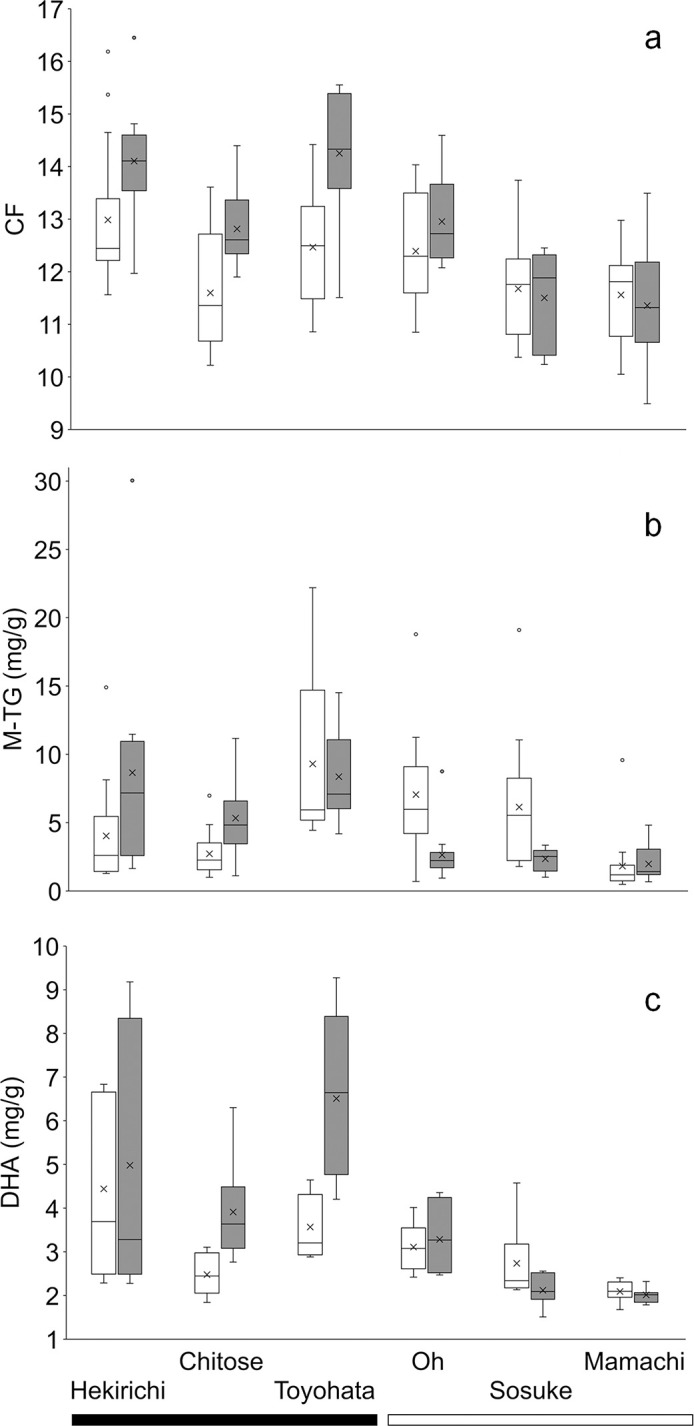
Box plots of (a) condition factor (CF), (b) muscle triglyceride (M-TG), and (c) DHA for brown trout sampled before (white boxes) and during the peak (gray boxes) of the chum salmon fry stocking period in stocked (Hekirichi, Chitose, and Toyohata rivers; black bar) and unstocked (Oh, Sosuke, and Mamachi rivers; white bar) rivers. Crosses and lines in the middle of boxes show means and medians. Box upper and lower boundaries show 75th and 25th percentile lines, respectively. Bars extend to 1.5 times the box height above and below the boxes. Small circles show outlier data points.

Timing × RT (river type) was significant in all linear mixed models for CF, M-TG, and DHA, indicating higher nutritional status during the peak stocking period than before the stocking period in stocked rivers (Table 3; Fig 4). Before the stocking period, log_10_FL × release was not significant, meaning that there was no difference in the slope of log_10_FL relative to log_10_BW_T_ between stocked and unstocked rivers (*F* = 0.0007, *p* = 0.979; S2 Fig in S2 File). By contrast, log_10_FL × release was significant during the peak stocking period (*F* = 5.228, *p* = 0.026; S2 Fig in S2 File), and the estimated slope in stocked and unstocked rivers was 3.07 and 2.90, respectively. FL was also significant in the M-TG and DHA models (Table 3), with higher values of both nutritional indices at larger FL (S3 Fig in S2 File). Additionally, there was a significant positive correlation between M-TG and DHA regardless of river type or timing (Table 4), and M-TG and DHA were positively correlated with CF in stocked rivers.

**Table 3:** Results of selected linear mixed models testing the effects of log_10_-transformed fork length (log_10_FL), timing (pre- or peak stocking period), river type (stocked or unstocked), and their interaction terms on condition factor (CF), muscle triglyceride (log_10_M-TG), and log_10_DHA of brown trout.

	d.f._numerator_	d.f._denominator_	*F*	*p*
**CF**				
Timing	1	136.25	15.32	<0.001
River type (RT)	1	4.02	3.98	0.117
Timing × RT	1	136.25	13.05	<0.001
**Log**_**10**_ **M-TG**				
Log_10_FL	1	137.44	9.91	0.002
Timing	1	135.23	1.07	0.303
RT	1	3.97	2.60	0.183
Timing × RT	1	135.18	18.03	<0.001
**Log**_**10**_ **DHA**				
Log_10_FL	1	76.02	10.61	0.002
Timing	1	75.05	8.25	0.005
RT	1	4.01	7.32	0.054
Timing × RT	1	75.02	14.39	<0.001

**Table 4:** Coefficients and *p*-values of Pearson’s correlations among muscle triglyceride (M-TG), DHA, and condition factor (CF) in brown trout before chum salmon fry stocking and during the peak of fry stocking in stocked and unstocked rivers.

River type	Before or peak	M-TG vs. DHA		M-TG vs. CF		DHA vs. CF	
		*r*	*p*	*r*	*p*	*r*	*p*
Stocked	Before	0.44	0.045	0.40	0.015	0.67	<0.001
	Peak	0.67	<0.001	0.36	0.045	0.42	0.055
Unstocked	Before	0.56	0.008	0.07	0.672	0.06	0.784
	Peak	0.62	0.003	0.18	0.291	0.47	0.032

## Discussion

Our results indicate that brown trout at the peak of the chum salmon fry stocking period tended to be better nourished in stocked rivers than in unstocked rivers. As previously reported in a number of rivers in Hokkaido [19], predation on chum salmon fry was observed in all stocked rivers, although predation pressure may vary among the rivers depending on the biotic (e.g., prey availability) and abiotic (e.g., water temperature and stream morphology) conditions in each stream. Chum salmon fry accounted for >60% of total MWW in the Chitose River throughout the stocking period. Brown trout are widely distributed throughout the Chitose River and prey on large numbers of chum salmon fry immediately after fry stocking, with one fish reported to have preyed on 481 fry within 1 day after stocking [29]. This suggests that the massive, pulsed supply of fry released into stocked rivers is an important food resource that quickly fills the stomachs of brown trout distributed over a wide area.

In the Hekirichi River, brown trout sampling for the peak of the stocking period occurred 19 days after the last fry release of the season. Because most chum salmon fry in Hokkaido rivers after April migrate to the sea within 10 days of release [27], it is likely that few fry remained in the Hekirichi River during the brown trout sampling, which is consistent with the low fry MWW observed. Other fishes and Amphipoda accounted for relatively large proportions of the MWW during the peak of the stocking period in the Hekirichi and Toyohata rivers, respectively. Although these results provide only a snapshot of brown trout diets during the sampling periods, they may reflect variations in food niche among individual salmonids [60]. For example, in the Hekirichi River a single brown trout preyed on 77 other fishes (other brown trout preyed on three or fewer other fishes), and in the Toyohata River three brown trout preyed on more than 10 Amphipoda.

All nutrient indices tended to be higher in the Chitose River than in the Mamachi River after March, when fry stocking began. CF also increased in the Mamachi River after March, but CF in the Mamachi River in May was at the same level as that in the Chitose River in March, and CF in the Mamachi River in June was at the same level as that in the Chitose River in April (Fig 3A). If we assume that the results from the Mamachi River reflect the normal recovery of CF among brown trout in unstocked rivers, brown trout in the Chitose River appear to have recovered their CF 2 months earlier than normal. Although salmonids are generally considered to be in good condition once their CF exceeds 14 [61], the mean and median CF in the present study exceeded 14 only in May and June in the Chitose River and during the peak of the stocking period in the Hekirichi and Toyohata rivers, suggesting faster recovery in stocked rivers.

During the stocking period, both M-TG and S-TG tended to be higher in stocked rivers than in unstocked rivers. The whole-body TG content of a well-fed chum salmon fry is 14‒23 mg/g [53], and the high TG observed in brown trout in the present study is likely to reflect the absorption of a large amount of TG from salmon fry. In particular, S-TG in March and April was much higher in the Chitose River than in the Mamachi River. Extremely high S-TG values (>1000 mg/dL) were observed in some relatively small individuals, possibly explaining the negative FL effect in the model. In wild brown trout from other regions, blood TG levels rarely exceed 500 mg/dL [e.g., 62‒64]. Our results suggest that S-TG increased rapidly in the Chitose River owing to heavy predation on chum salmon fry once stocking began in March and remained very high as stocking continued into April. M-TG increased further after May in the Chitose River, suggesting that TG eventually accumulated in the muscles. Moreover, the elevated IGF-1 in the Chitose River in May indicates that stored nutrients were being invested into growth, as has been suggested for white-spotted charr preying on masu salmon fry [65]. Increased DHA is also considered to be an effective indicator of nutritional improvement by predation on salmon fry [37, 50]; in the present study, this was especially notable in the Chitose and Toyohata rivers, where there was a large difference between pre- and peak stocking period samples. The positive correlation between M-TG and DHA suggests that M-TG was increased by predation on DHA-rich salmon fry in the stocked rivers rather than on aquatic insects or other invertebrates. In general, there is a positive correlation between muscle lipid content and CF in fish [66, 67]. In the present study, M-TG was correlated with CF in stocked rivers, indicating that CF improved for individuals that accumulated M-TG by preying on salmon fry. However, the observed trends were not consistent in all rivers (i.e., M-TG in the Toyohata River and DHA in the Hekirichi River) and showed considerable variation (Fig 4). These variations could reflect differences in seasonal prey availability among rivers and in the food niches of individual brown trout.

In the comparison of the six rivers, FL significantly affected multiple nutritional indices, likely because larger brown trout had a higher proportion of salmon fry in their diet during the stocking period. Piscivory in brown trout increases with body size [33, 68, 69], and in this study salmon fry were detected more often in the stomachs of large individuals. On the other hand, comparisons between the Chitose and Mamachi rivers showed no significant effect of FL for many indices. This could be because of the relatively small body size variance among the compared groups (range of mean FL: 19.9‒28.6 cm). Further study, including with captive experiments, is needed to determine how each index is affected by body size.

Our results demonstrate that brown trout restore their nutritional status through predation on stocked chum salmon fry. This suggests that chum salmon stocking may be helping to sustain brown trout populations to some extent by improving survival and growth. Not only non-native salmonids but also stocked hatchery salmonids are known to alter stream ecosystems [7, 24]. It would be interesting to investigate how the predator–prey relationship between non-native brown trout and stocked chum salmon fry modifies each impact on stream ecosystems. Meanwhile, our results highlight the potential for controversy among commercial fishermen and recreational anglers because valuable fisheries resources are being preyed upon by non-native species—prime targets of anglers—that are being fattened up as a result. At the very least, brown trout in stocked rivers can be considered more likely to maintain their populations through this predation; these rivers would therefore need to be given high priority as the targets of any brown trout removal programs.

To further clarify the interaction between the two species, it is necessary to determine whether the restoration of nutritional status observed in the present study leads to increased brown trout growth (as suggested by the IGF-1 results) and survival and ultimately to increased investment in reproduction as a “carry-over effect” [70]. In the Trinity River in the United States, non-native brown trout have been shown to prey on large numbers of stocked salmon juveniles, and it appears that the brown trout population there increased substantially after hatchery releases of salmon juveniles began in the 20th century [33]. In addition, brown trout with poor nutritional status are more likely to take on the anadromous form [46, 62, 63, 71], and the presence of suitable habitats in the Kerguelen Islands has been linked to a lack of range expansion via the ocean by brown trout there [72]. These reports suggest that massive stocking of salmon fry might even affect life history selection in brown trout, reducing the incidence of the anadromous form. Therefore, further research is required to elucidate the effects of massive stocking of chum salmon fry on brown trout at the population level. Such knowledge will be indispensable in developing more comprehensive river management measures, including hatchery programs and invasive species management.

## Supporting information

S1 File Supporting information—contains the supporting S1 and S2 Tables. (XLSX)

S2 File Supporting information—contains the supporting S1‒S3 Figs. (DOCX)
