# Supplementary material for: Massive stocking of chum salmon (Oncorhynchus keta) fry fattens non-native brown trout (Salmo trutta) in Hokkaido, Japan
Source: PLoS One. 2024 Jul 19;19(7):e0307552. doi: 10.1371/journal.pone.0307552 (PMC11259280; doi:10.1371/journal.pone.0307552)
Supplement: S2 File — (DOCX) [file pone.0307552.s002.docx]

**S1 Fig.** Relationships between fork length (FL) and true body weight (BW_T_, fish body weight excluding stomach content weight) in the Chitose (red) and Mamachi (blue) rivers from February to June. Each point indicates a brown trout. Dotted lines show regression lines; the corresponding formulae are shown in the figure.

**S2 Fig.** Relationships between fork length (FL) and true body weight (BW_T_, fish body weight excluding stomach content weight) in rivers before (left) and during the peak (right) of the chum salmon fry stocking period. Each point indicates a brown trout. Colors indicate sampling rivers, with warm colors corresponding to stocked rivers and cool colors to unstocked rivers. Dotted lines are regression lines for stocked (red) and unstocked (blue) rivers; the corresponding formulae are shown in the figure.

**S3 Fig.** Relationships between fork length (FL) and muscle triglyceride (M-TG; upper) and docosahexaenoic acid (DHA; lower) in rivers before (left) and during the peak (right) of the chum salmon fry stocking period. Each point indicates a brown trout. Colors indicate sampling rivers, with warm colors corresponding to stocked rivers and cool colors to unstocked rivers. Dotted lines are significant regression lines generated by using linear mixed models of stocked (red) and unstocked (blue) rivers; the corresponding formulae are shown in the figure.
